# Studying the rigidity of red blood cells induced by Plasmodium falciparum infection

**DOI:** 10.1038/s41598-019-42721-w

**Published:** 2019-04-19

**Authors:** Apurba Paul, Ghania Ramdani, Utpal Tatu, Gordon Langsley, Vasant Natarajan

**Affiliations:** 1 Department of Physics, Indian Institute of Science, Bangalore, 560012 India; 2 Inserm U1016, CNRS UMR8104, Cochin Institute, Paris, 75014 France; 3 Department of Biochemistry, Indian Institute of Science, Bangalore, 560012 India

**Keywords:** Optical tweezers, Malaria

## Abstract

We study the effect of different chemical moieties on the rigidity of red blood cells (RBCs) induced by *Plasmodium falciparum* infection, and the *bystander effect* previously found. The infected cells are obtained from a culture of parasite-infected RBCs grown in the laboratory. The rigidity of RBCs is measured by looking at the Brownian fluctuations of individual cells in an optical-tweezers trap. The results point towards increased intracellular cyclic adenosine monophosphate (cAMP) levels as being responsible for the increase in rigidity.

## Introduction

Malaria remains a global health burden^1,2^, and can even result in death of infected patients. This is particularly true among children, because their immune system is not so well developed. The pathogenesis of the disease is caused by the red blood cells (RBCs) becoming rigid^3^, which prevents them from squeezing through narrow capillaries and carrying life-giving oxygen to tissues. In earlier work^4^, we studied the properties of single RBCs trapped in an optical-tweezers trap, and showed that there was an increase in the corner frequency (*f*_*c*_) from normal cells (nRBCs) to infected cells (iRBCs). Interestingly, we found a *bystander effect*, in which hosting and non-hosting RBCs showed the same change in properties.

In this work, we study the bystander effect in detail by adding three different chemical moieties to the culture medium. The results are preliminary, but suggest that the increased rigidity is caused by an increase in intracellular cAMP levels. cAMP was chosen as a candidate because previously published experiments have shown that infected RBCs have much higher cAMP levels compared to non-infected RBCs^5^. The increase in cAMP levels being responsible for the increased rigidity is also consistent with a model presented by some of us^6^, where the rise in intracellular cAMP levels activates protein kinase A (PKA) to phosphorylate RBC cytoskeletal proteins.

## Materials and Methods

### Optical-tweezers setup

**Figure 1:**
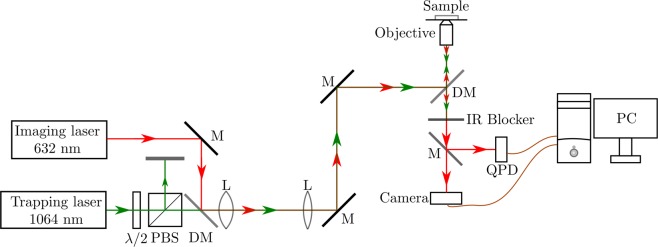
Schematic diagram of the optical-tweezers setup. Figure key: *λ*/2–halfwave retardation plate; PBS–polarizing beam splitter; DM–dichroic mirror; M–mirror; L–lens; QPD–quadrant photo-detector.

The setup for optical tweezers, shown schematically in Fig. 1, is the same as used in our earlier work^4,7^, and is reproduced here for completeness. It is based on a Zeiss inverted microscope having a high-power lens (comprising of a 100×, 1.4 NA, oil-immersion objective). The trapping beam is an Nd:YAG laser having a wavelength of 1064 nm and a maximum power of 500 mW. The actual power going into the experiment is controlled using a combination of a half-wave (*λ*/2) retardation plate and polarizing beam splitter (PBS). The trapping beam is mixed with an imaging beam, which consists of a HeNe laser with a wavelength of 632 nm and power of 5 mW. The trapping beam is mixed with the imaging beam on a dichroic mirror (DM), and the mixed beams are imaged on to the back plane of the objective.

The reflected beam from the trapped particle is used to track its position. The reflected trapping beam is blocked with an IR blocker, and only the red imaging beam is used for this. The position is measured using a quadrant photo-diode (QPD), whose output is acquired by a data acquisition (DAQ) card sitting on a computer slot. Brownian fluctuations corresponding to thermal motion of the trapped particle are acquired by the QPD, and analyzed using LabView software from National Instruments (NI).

### Sample preparation

The control experiments were done with a culture of normal cells (nRBCs), prepared according to the procedure given in Paul *et al*.^4^. Briefly, O-positive blood from a sample was centrifuged at 2000 rpm for 15 min, and a pellet of RBCs obtained. It was washed and suspended in 1× phosphate buffered saline (PBS) solution for the measurement.

Infected cells (iRBCs) were studied using the *Plasmodium falciparum* laboratory strain 3D7, cultured according to the standard protocol using the candle-jar method^8^. The strain was cultured in a Roswell Park Memorial Institute medium (RPMI-1640) in O-positive blood.

The bystander effect was studied by adding different chemical moieties to the culture medium. This was achieved by first dissolving the chemicals in a sterile dimethyl sulfoxide (DMSO) medium for 30 min, and storing them in a 10 µl at −20 °C. They were then added to the culture medium at a concentration of 5 µmol, and incubated for 30 min at 37 °C. After incubation, RBCs were removed and washed twice in 1 × PBS; then suspended in 1 × PBS for the measurement.

The nRBCs were obtained from a human blood sample got from a local blood bank. iRBCs were obtained by culturing the same sample. Since the sample was from a blood bank, the donation was done by a volunteer with complete awareness of the relevant guidelines for blood donation.

## Results

The experiment was designed to study the change (if any) in the mean corner frequency after incubation with different chemical moities, for both normal and infected RBCs. This was done by measuring *f*_*c*_ for 50 individual RBCs trapped in the optical-tweezers trap. The mean *f*_*c*_ (${\bar{f}}_{c}$) and standard deviation (*σ*) were calculated for each distribution. The standard error in the mean is $\frac{\sigma }{\sqrt{N}}$, where *N* = 50 is the number of points in the distribution.

The following three chemical moieties were added to the culture medium to test for the bystander effect.

1. Diamide, which decreases deformability of rabbit RBCs^9^, was used as a positive control.
2. cAMP levels were *increased* using a membrane permeable analogue called dibutyryladenosine cyclic mono-phosphate sodium salt, and denoted by db-cAMP.
3. cAMP levels were *decreased* by adding an inhibitor to cluster of differentiation 73 (Ecto-5′-nucleotidase) (CD73). CD73 is an ecto-5′-nucleotidase that converts AMP to adenosine^6^.

**Figure 2:**
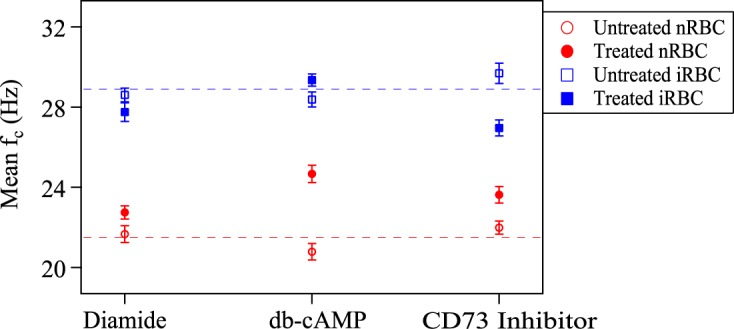
Mean *f*_*c*_ for nRBCs and iRBCs with and without inhibitor treatment. The error bar for each point represents 1*σ* error in the mean. The dashed lines represent the average value over 3 days.

The results are presented in Fig. 2. The experiments with non-treated RBCs are nominally the same; the values are different only because they were done on different days with the difference representing day-to-day variation in the experiment. As expected, the values are not very far from the dotted lines, which represent averages over the 3 days. The experiments were done on different days so that comparison with and without the chemicals could be done on the same day. The increase in average value from nRBCs to iRBCs shows that the present results are consistent with our earlier findings^4,7^. Most note worthy is that incubation with db-cAMP of nRBCs shows the maximum increase in ${\bar{f}}_{c}$ and half way to that of iRBCs, suggesting that cAMP mediates the bystander effect.

In order to highlight the statistical significance of the above analysis, we calculated the p-value for each pair of distributions, consisting of incubation with and without the chemicals. This was done for both nRBCs and iRBCs. Two distributions are considered significantly different if their p-value is less than 0.05. The p-value calculations are summarized in Table 1. As seen, the p-value is smallest for nRBCs treated with db-cAMP, which signifies the largest change in the two distributions. While incubation with the CD73 inhibitor also shows a significant difference (for both nRBCs and iRBCs), the change is not as big as the one with db-cAMP for nRBCs. The p-values for the other 3 cases are near 0.05, indicating that they are not significant.

**Table 1:** p-value for different combinations.

Type 1	Type 2	p-value
nRBC + Diamide	nRBC − Diamide	0.05
iRBC + Diamide	iRBC − Diamide	0.05
nRBC + db-cAMP	nRBC − db-cAMP	4.0 × 10^−19^
iRBC + db-cAMP	iRBC − db-cAMP	0.047
nRBC + CD73 Inhibitor	nRBC − CD73 Inhibitor	1.7 × 10^−6^
iRBC + CD73 Inhibitor	iRBC − CD73 Inhibitor	5.6 × 10^−5^

Thus, consistent with the results in Fig. 2, we conclude that our study indicates that cAMP mediates the bystander effect.

## Discussion

The clinical signs of pernicious human malaria stem from the invasion and development of *P. falciparum* parasites within RBCs. Infection is accompanied by major changes in iRBCs that affect their lipid composition and plasma membrane deformability^10^. This contributes to both sequestration of iRBCs, and the increased rigidity of iRBCs leads to cerebral malaria, the most severe pathological complication^10,11^. Non-infected RBCs also release adenosine triphosphate (ATP) in response to mechanical deformation that occurs as they flow from arteries to veins and capillaries, and pass through the slits in the spleen^12,13^. During its intra-RBC development, *P. falciparum* makes iRBCs appear older and leach ATP and, as non-infected RBC ATP levels are high, when the RBC plasma membrane is damaged, lysed, or traversed by *Plasmodium* parasites during invasion, extracellular ATP levels can increase substantially^14–16^.

Conditioned media from cultured iRBCs when added to non-infected RBCs render their plasma membrane more rigid implying that iRBCs secrete into the media a metabolite(s) that has both autocrine and *in trans* effects on RBC plasma membrane deformability; a phenomenon we termed the bystander effect^4^, and have hypothesized that it corresponded to released ATP, or its breakdown products adenosine and inosine^6^. Ectonucleotidases are expressed on the RBC surface where cluster of differentiation 39 (Ectonucleoside triphosphate diphosphohydrolase-1) (CD39) can hydrolyze ATP and adenosine diphosphate ADP to AMP that gets converted to adenosine by CD73^17^. Extracellular adenosine can signal through adenosine A2A receptor (ADORA2A) and adenosine A2B receptor (ADORA2B) G protein coupled receptors to activate adenylate cyclases and increase intracellular cAMP levels; for review, see Zhang and Xia^18^. We therefore tested that the changes in membrane deformability induced by the bystander effect are derived from the breakdown of ATP leading to an increase in intracellular cAMP levels of both iRBCs and non-infected RBCs.

The results presented in Fig. 2 show that the increase in average value from nRBCs to iRBCs are consistent with our earlier findings^4,7^. *P. falciparum*-infected RBCs are known to have higher basal cAMP levels compared to non-infected RBCs^19–21^, and as a consequence the addition of exogenous db-cAMP to nRBCs leads to a lower overall change in cAMP levels in nRBCs and hence a less pronounced effect on plasma membrane deformability. Thus, we conclude that cAMP is the effector molecule of the bystander effect, and when combined with the changes observed upon CD73 inhibition it argues that the theoretical model of how the bystander effect might function is actually operational on nRBCs and iRBCs^22^. In this model, iRBCs release ATP that is converted to AMP by CD39, AMP to adenosine by CD73, and adenosine signals via the ADORA2B receptor on RBCs to raise intracellular cAMP levels. The rise in cAMP activates PKA to phosphorylate RBC cytoskeletal proteins^23,24^, hence changing the rigidity of the RBC plasma membrane. This change in rigidity is reflected in the observed alterations in mean corner frequencies.

This finding is also consistent with the fact that the addition of cAMP to nRBCs increases their adhesion to fibronectin. This is an independent observation about the role of cAMP in causing the bystander effect, and was done in the laboratory of two co-authors (GR and GL) in France. It is hence presented in a Supplementary file.

## Conclusions

In summary, we examined changes in properties of the plasma membrane of RBCs infected with the malaria-causing parasite–*P. falciparum*. RBC plasma membrane deformability was measured at room temperature by following Brownian fluctuations of single RBCs held in an optical-tweezers trap. To determine the nature of the bystander effect, different chemical moieties were added to nRBC and iRBC culture media to see if they altered their plasma membrane deformability. Our results suggest that the bystander effect is mediated by extracellular adenosine stemming from degradation of AMP. Extracellular adenosine then is the bystander molecule capable of changing plasma membrane deformability–*in cis* on iRBCs and *in trans* on nRBCs. cAMP is the effector molecule for this because it activates PKA to phosphorylate plasma membrane cytoskeletal proteins, so changing plasma membrane deformability.

The results are preliminary, because only one concentration of db-cAMP was tried, while in future work we plan to use different concentrations to pinpoint the exact level of cAMP needed for the bystander effect.

## Supplementary information

Studying the rigidity of red blood cells induced by Plasmodium falciparum infection
