# Supplementary material for: Studying the rigidity of red blood cells induced by Plasmodium falciparum infection
Source: Sci Rep. 2019 Apr 19;9:6336. doi: 10.1038/s41598-019-42721-w (PMC6474899; doi:10.1038/s41598-019-42721-w)
Supplement: Supplementary file 1 — Studying the rigidity of red blood cells induced by Plasmodium falciparum infection [file 41598_2019_42721_MOESM1_ESM.pdf]

Studying the rigidity of red blood cells induced by *Plasmodium falciparum* infection

Apurba Paul[1], Ghania Ramdani[2], Utpal Tatu[3], Gordon Langsley[2], and Vasant Natarajan[1,*]

[1]Department of Physics, Indian Institute of Science, Bangalore-560012, India
[2]Inserm U1016, CNRS UMR8104, Cochin Institute, Paris 75014, France
[3]Department of Biochemistry, Indian Institute of Science, Bangalore-560012, India
[*]vasant@physics.iisc.ernet.in

Assay showing that the addition of cAMP to nRBCs increases their binding to fibronectin

Preparation of RBCs with cAMP

Human RBCs were obtained by venipuncture from EFS Rungis (France), centrifuged at 1500 rpm, and washed three times in and resuspended with complete RPMI 1640. nRBCs and iRBCs of *P. falciparum* 3D7 clone were cultivated under identical conditions. The procedure for stimulation of cAMP with drug treatment is as follows. RBC count was carried out and cells were diluted to 10^4 in 1 ml RPMI. The cAMP analogue $\mathrm{N6}, 2' - \mathrm{O} - \mathrm{Dibutyryladenosine}\ 3', 5' - \mathrm{cyclic\ monophosphate}$ (Sigma D0627) was used at 50 µm concentration for 30 min at 37 °C. Untreated RBCs were used as negative control. After treatment, all tubes were centrifuged at 2000 rpm and the supernatant removed.

Adhesion assay

A 96 well plate was coated with fibronectin (Sigma F-0895 1 mg/ml), $2\,\mathrm{mg/cm^2}$ (7 µg in 100 µl per well) diluted in double distilled water. The plate was left overnight at 4 °C. Wells were then washed twice with 100 µl washing buffer (0.1% BSA in basal medium of RPMI). 100 µl of blocking buffer (0.5% BSA in basal medium RPMI) was added to each well and left for 1 hour at 37 °C. All wells were then washed twice with washing buffer. The plate was left to chill on ice while cells were prepared. Once cells were prepared, they were washed once with PBS and resuspended at 1×104 cells in complete RPMI, and then added at 100 µl per well. The plate was incubated for 1 hour at 37 °C, 5% CO_2. Next, non-adherent cells were removed and each well was given three washes with 100 µl of washing buffer. Cells were then fixed with 100 µl paraformaldehyde (PFA) 4% for 10 min at room temperature. The plate was then given one further wash with washing buffer and stained with 100 µl crystal violet (5 mg/ml) for 10 min at room temperature. All wells were then washed extensively with distilled water and the plate was turned upside down to air dry. Once dry, cells were solubilised with 100 µl 2% sodium dodecyl sulphate (SDS) and 2% ethanol for 30 min at room temperature before reading at optical density (OD) 595 nm.

Images

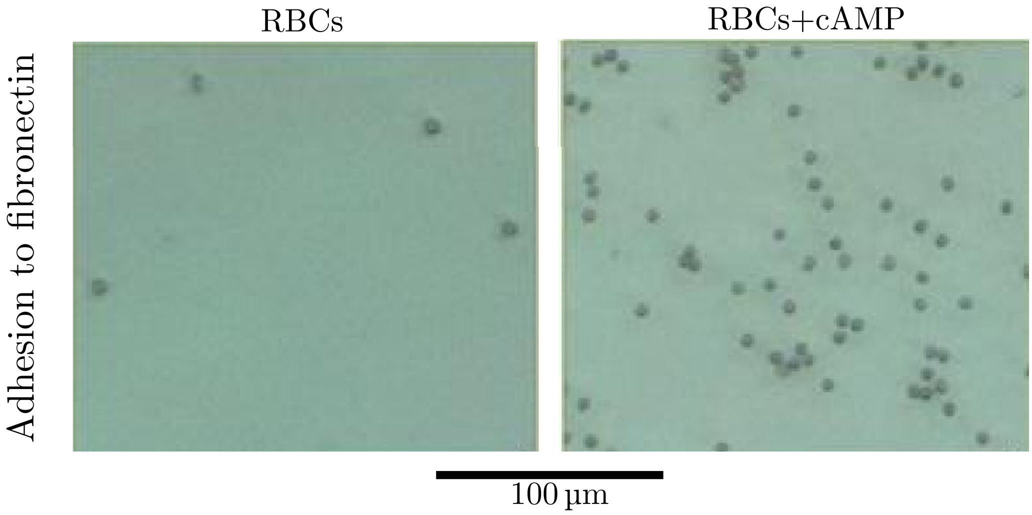

The number of RBCs within the field of view increases dramatically when cAMP is added.
